# The effects of interactive video games combined with LEGO game therapy on social anxiety in rural left-behind children

**DOI:** 10.3389/fpsyg.2024.1423755

**Published:** 2024-12-20

**Authors:** Dianhui Peng, Xinbo Wu, Yaqi Yang, Xiaolin Li, Anni Shu, Junwen Liang, Zengying Tu, Le Liu, Qian Yang, Weixin Dong, Chunxia Lu

**Affiliations:** ^1^Department of Sport Education, Hunan Normal University, Changsha, China; ^2^College of Sports and Art, Shandong Sport University, Jinan, China

**Keywords:** rural left-behind children, interactive video games, LEGO game therapy, social anxiety, social interaction

## Abstract

**Background:**

China has a significant population of left-behind children, defined as those whose parents have migrated away from their home for at least six months. This situation arises when one or both parents leave to seek work in urban areas. Among the various challenges faced by left-behind children, social anxiety has emerged as one of the most pressing issues.

**Objective:**

This study aims to deeply explore the impact of interactive video games and LEGO games on the social anxiety of rural left-behind children, with the aim of providing a more comprehensive and effective solution to the mental health problems they encounter.

**Methods:**

Eighty-four rural left-behind children were randomly assigned to four groups: interactive video game group, LEGO game group, combined intervention group, and the control group, using a single-blind randomized controlled experiment. All participants underwent the intervention three times a week for 12 weeks. Social anxiety levels were assessed at three points: before the intervention (T_0_), immediately after the intervention (T_1_), and 6 weeks post-intervention (T_2_).

**Results:**

The effects of different intervention strategies on the social anxiety of left-behind children showed significant group-time interaction effects. As the intervention progressed, the total score of social anxiety and the scores of various dimensions among participants in different intervention groups showed a gradual decreasing trend. The combined intervention group scored significantly lower at T_1_ and T_2_ than both the interactive video game group and the LEGO group.

**Conclusion:**

Our findings indicate that social anxiety in left-behind children can be significantly mitigated through both LEGO therapy and interactive video games, with a combined approach yielding the most pronounced effects. These results underscore the importance of a multifaceted intervention strategy that addresses the unique social needs of this vulnerable population. Importantly, our study suggests that effective interventions should not only focus on symptom reduction but also foster environments that enhance social skills and build confidence. Future research should investigate the scalability of these interventions across diverse contexts and their potential integration into existing mental health services to better support left-behind children.

## Introduction

In the past three decades, China has witnessed rapid economic growth alongside accelerated urbanization, yet significant economic disparities persist between urban and rural areas. This imbalance has driven numerous rural residents to migrate to cities in pursuit of better job opportunities, leaving their children behind in rural areas, often under the care of grandparents or other relatives. These children, known as ‘left-behind children (LBC)’, number 41.77 million according to the seventh national population census conducted by the National Bureau of Statistics of China in 2020, representing 14.03 percent of the nation’s child population. Separated from their parents for extended periods, LBC inhabit an environment where their physical and mental needs may go unmet (Shi et al., 2023). Comparative studies with non-LBC suggest a heightened susceptibility to psychological challenges (Wang et al., 2018), including emotional instability, social withdrawal, loneliness, and feelings of inferiority. These issues not only hinder the growth and development of LBC but also pose a potential threat to social harmony and stability. Social anxiety disorder is a significant issue among LBC, with a detection rate of 36.1%, compared to just 20.2% among non-LBC (Li et al., 2019). For the unique cohort of LBC, enduring prolonged absence of parental companionship and emotional nurturing, their social well-being often heavily relies on peer interaction and support (Xu et al., 2024). However, due to deficits in social skills, many LBC exhibit a tendency towards withdrawal and avoidance of peer communication (Liu, 2022), exacerbating their symptoms of social anxiety (Liu and Xu, 2020). Social anxiety manifests as heightened anxiety, nervousness, or fear in specific interpersonal situations, characterized by nervous demeanor, fearfulness, excessive apprehension, aversion to eye contact, discomfort, and avoidance during social interactions (Morrison and Heimberg, 2013). Prolonged separation from parents deprives LBC of direct emotional support and security, contributing to emotional loneliness and helplessness (Zhang and Shi, 2021). This emotional deficit may lead LBC to display nervous, insecure, and avoidant behaviors in social contexts, increasing the risk of social anxiety disorder (Zhang et al., 2024). Social anxiety, as a chronic condition, not only exacerbates over time but also elevates the risk of other severe mental health issues, such as moderate to severe insomnia, suicide attempts, substance abuse, and depression (Raffray et al., 2011; Duffy et al., 2020; Kalin, 2020; Single et al., 2022). Thus, advancing treatment modalities for LBC with social anxiety disorder is imperative for promoting their healthy development.

In recent years, research has highlighted the importance of fostering social interactions and collaborative experiences in interventions aimed at addressing social anxiety in children. Evidence suggests that increasing opportunities for positive social interactions among peers can alleviate symptoms of social anxiety (Cordier et al., 2021). With this in mind, the current study explores two distinct, peer-focused approaches—Interactive Video Games (IVG) and LEGO Game Therapy (LGT)—to reduce social anxiety in LBC by fostering structured, collaborative environments. In this study, IVG are employed as a means to create structured, peer-based social experiences. The games encourage active social engagement, helping children develop social skills, build relationships, and reduce anxiety through repeated interactions in a safe, controlled virtual environment (De Troyer, 2017; Schakel et al., 2020). While IVG encompass a variety of game modalities, the intervention focuses on games that require interaction between players, thereby promoting the development of social competencies. Previous studies have shown that engaging in IVG can improve decision-making and cognitive abilities across various age groups, including older and younger adults (Reynaldo et al., 2021), while also fostering positive emotional experiences, enhancing self-esteem, promoting well-being, and reducing anxiety (Pallavicini and Pepe, 2020). Instead of classifying games strictly as cooperative, competitive, or exergames, this study emphasizes the social interactions that can occur in various gameplay types. Cooperative games involve players working together toward a shared goal, while competitive games involve players competing against each other. Exergames, which promote physical activity, can also be cooperative or competitive, depending on their design. What sets the games used in this study apart is their ability to facilitate social interactions among children, whether through collaboration or competition, thereby helping them develop important social skills in an engaging way. A recent study comparing these game types found that cooperative gameplay was more effective in reducing anxiety and fostering verbal communication among children (Nekar et al., 2023). Additionally, LGT has become a widely utilized method in recent years for enhancing children’s social and communication skills (Wright et al., 2023). This form of play therapy involves peer-based social interventions, wherein participants collaborate in social division of labor, construct LEGO models, and engage in verbal, visual, and non-verbal social communication (LeGoff, 2004). Through this process, individuals develop crucial social skills such as communication and empathy. Lego therapy is frequently employed in the treatment of children with autism spectrum disorder (ASD) and has shown efficacy in reducing parent-reported separation anxiety and self-reported social anxiety among children with ASD (Choy et al., 2024). In addition to this, LEGO games are used to improve the communication and social skills of children and teenagers of all ages, allowing children to experience a sense of mastery and achievement, and making it easier for them to explore, build and express themselves (Barr et al., 2022; Vusio et al., 2022). Given the complexity of social anxiety among LBC, researchers have increasingly advocated for combination interventions, which integrate multiple therapeutic techniques to address the multifaceted needs of children. Combination approaches, which often blend structured virtual interactions with hands-on, physical activities, are considered particularly effective for students with diverse learning styles and social skill levels (Mallek et al., 2024). By incorporating both IVG and LGT, this study seeks to leverage the unique strengths of each approach—IVG’s ability to engage children in immersive, interactive digital environments, and LGT’s emphasis on physical, collaborative play. This dual approach is designed to provide both virtual and real-world platforms for social skill-building, allowing LBC to build confidence, competence, and meaningful peer connections across different settings. The combined approach may yield more comprehensive results, as it addresses social anxiety from multiple angles, offering a dynamic range of interactions that cater to children’s varied preferences and needs.

Despite ongoing efforts, the most effective methods for reducing social anxiety in LBC remain elusive, with limited evidence available regarding specific intervention strategies. Exploring diverse therapeutic approaches can enhance intervention outcomes by addressing multiple aspects of a child’s social and emotional experience. Virtual games capture children’s attention with interactive features, while hands-on LEGO play supports direct, physical social interaction, accommodating different preferences and learning styles (Sayis et al., 2022).These approaches encourage a variety of social interactions, potentially enhancing engagement and supporting the development of social skills. However, few studies have examined how these interventions impact social anxiety among LBC. Therefore, this study aims to investigate whether different types of social interactions can reduce social anxiety in LBC. By fostering collaboration, emotional connection, and skill development, these interventions have the potential to address the core challenges associated with social anxiety and promote healthier social and emotional development in this vulnerable population.

## Methods

### Study design

This study was designed as a experimental design conducted by a research team comprising an experienced professor with expertise in sports rehabilitation and clinical psychology, three postgraduate students in sports science, and three graduate students in psychology. The study employed a rigorous approach to data collection throughout its implementation. Initially, baseline measurements were taken for all participants to record their social anxiety score prior to the intervention. Subsequently, after 12 weeks of intervention, the same measurement process was repeated to evaluate the intervention’s effectiveness. Finally, a follow-up assessment was conducted six weeks after the intervention concluded to assess the sustainability of the intervention’s effects. The study received approval from the Biomedical Research Ethics Committee of Hunan Normal University (Approval No. 2022 No. 309) and adhered to the ethical principles outlined in the Declaration of Helsinki for research involving human subjects. Written informed consent was obtained from all participants prior to their enrollment in the study.

### Procedures

#### Study setting

The study was conducted in a rural primary school (Huangdu School) located in Shaodong City, Hunan Province. The participants were selected from 8 different classrooms within the same school. Prior to the intervention, the children were familiar with each other as they attended the same school, though they were distributed across different classrooms. This existing familiarity allowed for a baseline level of social anxiety before the intervention began.

#### Group assignment

Participants were randomly assigned to either the treatment groups (IVG, LGT and combined intervention group) or the control group. Children in the treatment groups were pulled out of their regular classroom activities for the intervention sessions, which were held four times per week. Each session took place in a designated intervention room, separate from the regular classroom setting, to create a focused and consistent environment for the therapy. The control group continued their regular school activities without any intervention. The treatment groups followed their standard school curriculum when not participating in the study, ensuring that their academic activities were minimally disrupted.

### Participants

Before starting the study, researchers used G*Power 3.1.9.7 software to determine the sample size. It was estimated that a minimum of 17 cases per group would be necessary, based on a power value of 0.85, an alpha level of 0.05, and an effect size of 0.44. The sample included 84 LBC, with 46 identifying as male and 38 as female. Their ages ranged from 9 to 11 years, with a mean age of 10.405 years (SD = 0.873). Hunan Province is known for its high number of LBC, particularly in Shaodong City. The inclusion criteria were as follows: (1) LBC in rural areas who have experienced parental separation for more than 2 years; (2) aged between 9 and 11 years old; (3) self-reported total social anxiety test score of ≥8; (4) no intake of antidepressant in the past 3 months; (5) absence of serious physical ailments or mental disorders; (6) no prior participation in LGT or IVG training. Exclusion criteria included: (1) gross or fine motor disorders; (2) receiving medication or psychological counseling for mental health issues. This study recruited a total of 94 LBC. After excluding 10 participants who did not meet the inclusion criteria, the remaining 84 participants were sorted alphabetically by last name and randomly assigned to four equal-sized groups (n = 21 each) using an online randomization tool (http://www.randomizer.org): IVG group, LGT group, combined intervention group, and the control group (CG). During the intervention, all of the LBC participated, with no absences reported.

### Intervention components

#### Interactive video game

The entire intervention spanned 12 weeks, comprising three 45-min sessions per week. Participants formed teams of 7 based on personal preferences for game partners. Prior to each session, a 5 to 10-min warm-up session, led by a student assistant, was conducted. During this period, the research assistant ensured the IVG classroom’s cleanliness, organized the teaching environment, and verified equipment functionality. The classroom accommodated two X-BOX 360 Kinect™ game consoles (Microsoft, Redmond, Washington, USA), facilitating multiplayer games for two to four children simultaneously. The Xbox 360 Kinect™ is a motion-sensing input device developed by Microsoft for use with the Xbox 360 video game console. While two to four children played the game, the other three to five children either watched, discussed strategies, or waited for their turn. The session facilitator kept everyone engaged by encouraging group discussions about the game and explaining the players’ in-game choices. However, children who were not actively playing might have experienced a different level of engagement compared to those playing at the moment. The facilitator also managed the order of play to ensure that each child had an equal opportunity to participate. In a 45-min session, each child received about 20 min of actual gameplay. These consoles were connected to projectors to ensure all participants had clear visibility of the gameplay. The intervention unfolded in two phases: during the initial six weeks, participants engaged with the “Adventures” mode on Kinect, which includes a variety of interactive games designed to promote physical activity and teamwork. This mode is characterized by its immersive environments and engaging narratives that require players to complete specific tasks or challenges. The five games included—"Fruit Ninja,” “Reflex Ridge,” “20,000 Leaks,” “River Rush,” and “Rally Ball”—were selected for their ability to encourage movement and collaboration among players. Each game was structured to gradually increase in difficulty, helping participants build their skills over time. Participants began with “Fruit Ninja,” which required quick reflexes to slice fruit while avoiding bombs, establishing a baseline of engagement and motor skills. The sequence of games was designed to promote increasing levels of coordination and teamwork, with “Rally Ball” concluding the Adventures phase as a culmination of the skills learned. Subsequently, in the following six weeks, participants transitioned to the second phase, involving Kinect’s “Sports” mode. This mode offered a different set of challenges, focusing on traditional sports simulations that provided a competitive element. The six sports simulation games—"Tennis Ball,” “Basketball,” “Skiing,” “Football,” “Badminton,” and “Boxing”—were carefully selected to promote teamwork and physical fitness. In this phase, participants began with “Tennis Ball,” which emphasized hand-eye coordination and strategic placement, before moving on to “Basketball,” where they practiced shooting and passing in a team setting. Each game was designed to build upon the previous skills learned, culminating in “Boxing,” which required coordination and reflexes.

#### Lego game therapy

Participants underwent a 12-week Lego play intervention, conducted thrice weekly, with each session lasting 45 min. The 21 participants were divided into three groups, each comprising two suppliers, two architects, and three engineers, overseen by a professionally trained therapist (student researcher). Throughout the intervention, the engineer delineated the required parts, while the parts supplier located the corresponding blocks, subsequently passing them to the architect for assembly under the engineer’s guidance. The building goals for each session were selected based on the participants’ developmental levels and interests, promoting creativity and teamwork. Examples of these goals included building a bridge, a vehicle, and a community center, all of which encouraged collaboration and problem-solving. Each goal was designed to be challenging yet achievable, providing a sense of accomplishment. At the start of each session, participants were informed of their building goal, and visual aids were used to show the desired outcome. Clear signals indicated when to change roles; for instance, after completing a significant part of the project, participants were encouraged to switch roles, allowing everyone to experience different functions within the team. Role changes occurred every 10 min or after specific tasks were completed, helping to keep everyone engaged and fostering a sense of shared responsibility. The therapist (student researcher) determined the developmental level of each group’s tasks by assessing the participants’ cognitive and social abilities throughout the intervention. While all groups had the same overall building goals, the complexity of the tasks varied to match each group’s developmental level. For example, one group might build a more complex bridge with advanced features, while another group focused on a simpler structure. This variation ensured that all tasks were suitable and challenging for the participants. Throughout the intervention, activity rules were prominently displayed on a bulletin board in the Lego therapy room, with strict adherence mandated for all participants. Examples of these rules included: ‘If you break it, you must fix it,’ ‘If you cannot fix it, seek assistance,’ and ‘If others are using it and you need it, ask first,’ among others. These rules aimed to instill positive habits and nurture a sense of community among participants. The process of group intervention is presented in Table 1.

**Table 1:** Lego game therapy intervention process and content.

Step	Time (min)	Content
1	0–5	Group members greet each other and introduce themselves, fostering a sense of familiarity and camaraderie. The therapist then acquaints the participants with the fundamental rules and gameplay mechanics of the Lego games.
2	5–20	The intervention progresses into role assignment and preliminary construction. Participants are assigned three distinct roles for the first time: supplier, architect, and engineer. Each role entails unique responsibilities and tasks, fostering collaboration to achieve the initial building goal within a 15-min timeframe. Upon successful completion of the task model, children enthusiastically applaud or exchange high-fives with one another.
3	20–40	The intervention progresses into role rotation and intensive collaboration. Throughout the next 20 min, participants will rotate through roles, ensuring each member experiences various responsibilities to bolster their sense of teamwork. Following the completion of the second and third task models, the students persistently expressed their joy and sense of accomplishment through celebratory gestures.
4	40–45	The therapist directs participants to tidy up their Legos and organize the activity area in an orderly fashion. Simultaneously, everyone bids farewell to each other, concluding this enriching group Lego therapy intervention on a positive note.

#### Combined intervention

The participants in this group engaged in a 12-week training program, meeting three times per week, which included sessions on IVG and LGT, as described above. Each session consisted of 45 min of interactive video game training, followed by a 30-min break, and concluded with 45 min of LEGO game training.

#### Control group

The control group comprised a subset of 21 rural LBC randomly selected from the study population. They did not receive any specific intervention during the 12-week study period but were monitored similarly to the intervention groups. The control group served as a reference to evaluate the effectiveness of the intervention strategies employed in this study.

### Measurement

First, basic information about the participants and their parents was collected, including general demographic information such as gender, age, parental marital status, parental outings, and parental frequency of returning home. Parental outings refer to the periods when parents are away from home engaging in activities outside the family environment, which may include work commitments, or social engagements. The Social Anxiety Scale for Children (SASC) for LBC was developed by La Greca (La Greca et al., 1988). Chinese scholar Wang made Sinicization revision (Wang et al., 1999). The scale is applicable to children and adolescents aged 7 to 16 and contains 10 questions (for example: I am afraid of doing something I have not done before in front of other children), including two dimensions of Fear of Negative Evaluation (FNE) and Social Avoidance and Distress (SAD). The score was scored on a three-level scale: never =0, sometimes =1, always =2, and the higher the total score, the more severe the social anxiety. According to the Chinese urban norm, a score of ≥8 is considered as social anxiety (Li et al., 2006). In this study, the Cronbach’s *α* of this scale was 0.892, and Cronbach’s α of all dimensions was greater than 0.7.

### Statistical analysis

Statistical analysis was conducted using GraphPad Prism (9th edition), and results were presented as mean ± standard deviation (M ± SD). Initially, differences in baseline characteristics among the four groups were assessed using either the chi-square test or one-way ANOVA. Subsequently, a repeated-measures ANOVA, employing a 4 (group: IVG group, LGT group, combined intervention group, control group) × 3 (time: pre-intervention, post-intervention, 6-week follow-up after intervention) design, was utilized to evaluate the effects of different intervention programs on the social anxiety levels of LBC. The significance level for all statistical tests was set at 0.05, with *p* < 0.05 (*) denoting statistical significance.

## Results

Table 2 outlines the basic characteristics, overall self-reported social anxiety scores, and their dimensions for the four groups of participants. The findings indicated no significant differences in baseline social anxiety scores and their respective dimensions among the four groups (*p* > 0.05).

**Table 2:** Characteristics of the participants in four different groups and baseline comparison.

Variable	Characteristic	CG (*n* = 21)	IVG (*n* = 21)	LGT (*n* = 21)	CIG (*n* = 21)	*F*/χ^2^	P
Gender	Male	12 (57.14)	11 (52.38)	11 (52.38)	12 (57.14)	0.06	0.98
	Female	9 (42.86)	10 (47.62)	10 (47.62)	9 (42.86)		
Age		10.57 ± 0.75	10.24 ± 0.89	10.19 ± 0.87	10.62 ± 0.97	1.35	0.26
Parental marital status	Married	14 (66.67)	13 (61.90)	14 (66.67)	12 (57.14)	0.87	0.46
	Divorced	4 (16.67)	5 (23.81)	5 (23.81)	5 (23.81)		
	Digamy	2 (9.52)	2 (9.52)	1 (4.76)	2 (9.52)		
	Widowed	1 (4.76)	1 (4.76)	1 (4.76)	2 (9.52)		
Parental outings	Father outing	10 (47.62)	9 (42.86)	13 (61.90)	11 (52.38)	0.42	0.74
	Mother outing	6 (28.57)	6 (28.57)	4 (19.05)	4 (19.05)		
	Parents outing	5 (23.81)	6 (28.57)	4 (19.05)	6 (28.57)		
Parental frequency of returning home	Once a week	2 (9.52)	2 (9.52)	2 (9.52)	2 (9.52)	0.10	0.96
	Once a month	3 (14.29)	4 (19.05)	2 (9.52)	2 (9.52)		
	Once a year	11 (52.38)	10 (47.62)	11 (52.38)	13 (61.90)		
	< Once a year	5 (23.81)	5 (23.81)	6 (28.57)	4 (19.05)		
SASC	Total	10.52 ± 1.99	11.14 ± 3.29	10.95 ± 3.29	11.00 ± 3.19	0.52	0.67
	FNE	6.14 ± 1.59	6.76 ± 2.05	6.71 ± 1.90	6.05 ± 2.36	1.53	0.21
	SAD	4.76 ± 1.30	4.81 ± 2.02	4.86 ± 1.42	5.05 ± 1.50	0.13	0.94

CG indicates control group, IVG indicates interactive video game group, LGT indicates LEGO game therapy group, CIG indicates combined intervention group.

**Figure 1:**
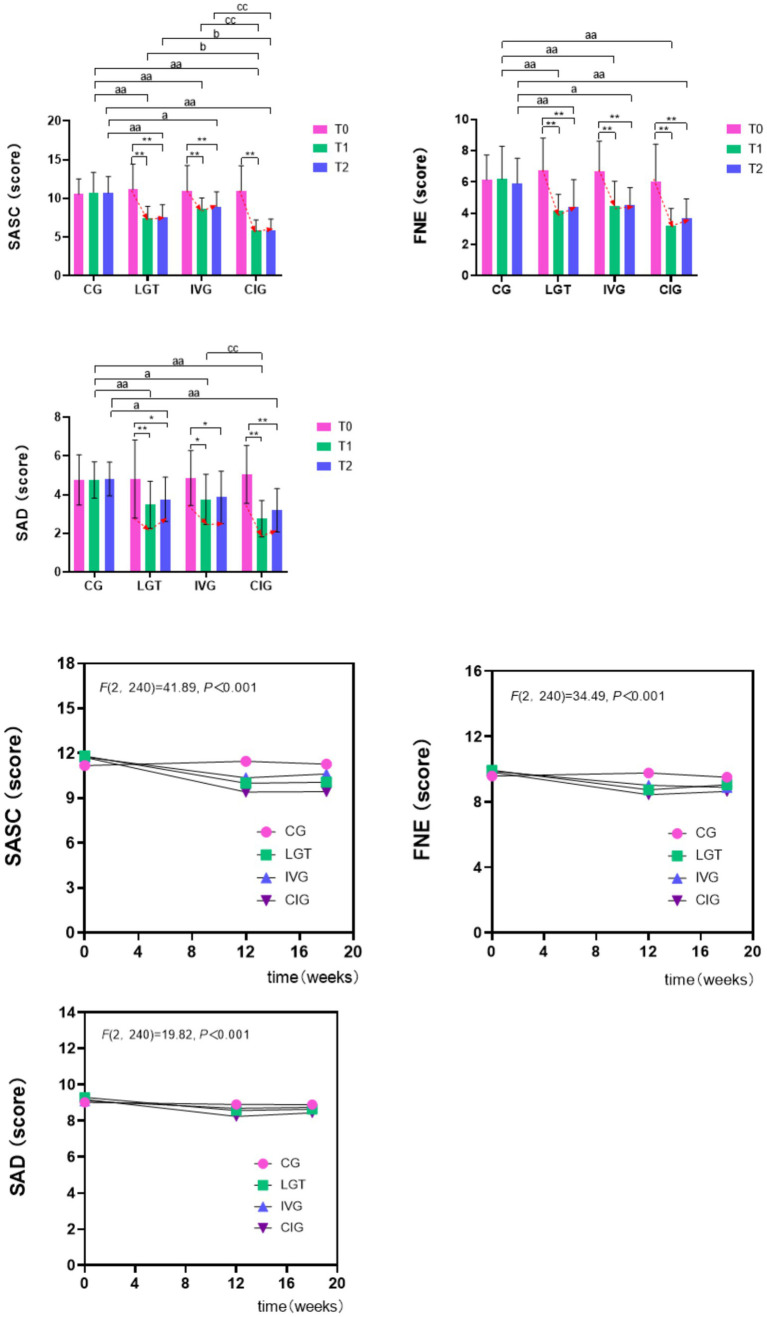
Effects of different interventions on SASC, FNE, SAD.

Table 3 presents detailed data on the total scores and two dimensions of social anxiety among the four groups at baseline and post-intervention, aiming to investigate the specific effects of different intervention strategies on social anxiety in LBC. Statistical analysis revealed a significant group-time interaction effect for the total score of SASC [*F*(6,80) = 8.55, *p* < 0.01, *η*^2^ = 0.24], indicating an interactive influence of intervention and time factors on social anxiety scores. Similarly, significant interaction effects were observed for the FNE factor [*F*(6,80) = 4.32, *p* < 0.01, *η*^2^ = 0.14] and SAD factor [*F*(6,80) = 4.16, *p* < 0.01, *η*^2^ = 0.14]. Given the presence of these interaction effects, further simple effect analyses were conducted. As depicted in Figure 1, social anxiety scores and scores on all dimensions exhibited a gradual decrease over intervention time across different intervention groups, with distinct variations observed among the groups.

**Table 3:** Effects of different interventions on SASC, FNE, SAD.

Index	Group	T_0_	T_1_	T_2_	*F/P* group	*F/P* time	*F*/*P* group*time
SASC	CG	10.52 ± 1.99	10.76 ± 2.61	10.67 ± 2.13	23.60/ <0.001^***^	41.89/ <0.001^***^	7.85/ <0.01^**^
	IVG	11.14 ± 3.29	7.43 ± 1.54aa^**^	7.57 ± 1.60aa^**^			
	LGT	10.95 ± 3.29	8.67 ± 1.39aa^*^	8.90 ± 1.95a^*^			
	CIG	11.00 ± 3.19	5.41 ± 1.40aabcc^**^	5.63 ± 1.41aabcc^**^			
FNE	CG	6.14 ± 1.59	6.19 ± 2.09	5.90 ± 1.61	11.90/ <0.001^***^	34.49/ <0.001^***^	3.72/ <0.01^**^
	IVG	6.76 ± 2.05	4.14 ± 1.06aa^**^	4.43 ± 1.72aa^**^			
	LGT	6.71 ± 1.90	4.48 ± 1.57aa^**^	4.52 ± 1.12a^**^			
	CIG	6.05 ± 2.36	3.19 ± 1.12aacc^**^	3.67 ± 1.24aa^**^			
SAD	CG	4.76 ± 1.30	4.76 ± 0.94	4.81 ± 0.87	8.08/ <0.001^***^	19.82/ <0.001^**^	3.19/ <0.01^**^
	IVG	4.81 ± 2.02	3.48 ± 1.21aa^**^	3.76 ± 1.14a^*^			
	LGT	4.86 ± 1.42	3.76 ± 1.30a^*^	3.86 ± 1.35^*^			
	CIG	5.05 ± 1.50	2.76 ± 0.94aac^**^	3.19 ± 1.12aa^**^			

a < 0.05, aa < 0.01, compared with CG; *b* < 0.05, compared with IVG; *c* < 0.05, cc < 0.01, compared with LGT; ^*^P < 0.05, ^**^P < 0.01, ^***^p < 0.001, compared with T_0_.

In our examination of time-related factors, we observed significant changes in the total score of SASC [*F*(2,80) = 47.83, *p* < 0.01, *η*^2^ = 0.37], FNE factor [*F*(2,80) = 40.14, *p* < 0.01, *η*^2^ = 0.33], and SAD factor [*F*(2,80) = 25.82, *p* < 0.01, *η*^2^ = 0.24] over time. Notably, the total score on the SASC, as well as scores for the FNE and SAD factors in Group CG, exhibited no significant change over time (*p* > 0.05). This finding suggests a relatively stable social anxiety status within this group throughout the intervention period. Conversely, participants in the IVG group, LGT group, and combined intervention group demonstrated significantly lower total social anxiety scores, FNE scores, and SAD scores at T_1_ and T_2_ compared to T_0_, with these differences achieving statistical significance (*p* < 0.05). These results suggest a positive impact of these interventions on reducing social anxiety levels.

In the analysis of group factors, significant effects were observed for the SASC total score [*F*(1,80) = 15.27, *p* < 0.01, *η*^2^ = 0.36], FNE factor [*F*(1,80) = 9.37, *p* < 0.01, *η*^2^ = 0.26], and SAD factor [*F*(1,80) = 5.51, *p* < 0.01, *η*^2^ = 0.17]. Initially, at baseline T_0_, no significant differences were found between intervention and control groups regarding SASC total score, FNE factor, and SAC factor (*p* > 0.05). However, during the intervention period, the control group exhibited significantly higher SASC scores compared to the other three groups (*p* < 0.05). Furthermore, the combined intervention group demonstrated significantly lower scores than the IVG group and LGT group at both T_1_ and T_2_ (*p* < 0.05). Regarding FNE scores, the control group also scored significantly higher than the other three groups at both T_1_ and T_2_ (*p* < 0.05). However, the combined intervention group scored lower than the Lego group at T_1_ (*p* < 0.05), with no significant difference between the two groups at T_2_. Concerning SAD scores, the control group showed significantly higher scores than the other three groups at T_1_ (*p* < 0.05). Notably, the only group with no significant difference from the control group at T_2_ was the LGT group. Additionally, the SAD score of the combined intervention group at T_1_ was significantly lower than that of the LEGO game group (*p* < 0.05). While the LGT group and the IVG group did not exhibit significant differences in total SASC score and scores in each dimension (*p* > 0.05), the IVG group scored lower than the LGT group at both T_1_ and T_2_.

## Discussion

This study represents the inaugural evaluation of the varying efficacy in ameliorating social anxiety among LBC through 12 weeks of IVG, LGT, and a combined intervention. The findings illustrate distinct alterations in social anxiety scores among the participants following each intervention. Notably, the combination intervention appeared to have the strongest effect on reducing social anxiety; however, it is difficult to attribute this improvement solely to increased peer engagement, as the combined intervention differed in duration and format from the other types.

The effectiveness of interactive video games in reducing social anxiety among LBC echoes findings from research involving typically developing children. In an era dominated by digital engagement, interactive video games, particularly those played with friends in person, serve as a prominent platform for children’s recreation and social interaction. In an era dominated by digital engagement, motion-based, cooperative interactive video games, particularly those played in person with friends, serve as a valuable platform for children’s recreation and social interaction. This study highlights the potential of these specific types of interactive games to reduce social anxiety among LBC, offering insights into their therapeutic value for this demographic. The games in our study were carefully selected for their emphasis on collaboration and physical activity, aiming to foster engagement and social skills through shared, real-time experiences. In this intervention, a total of 11 games are used (adventures mode: “Fruit Ninja,” “Reflex Ridge,” “20,000 Leaks,” “River Rush,” “Rally Ball”; sports mode: “Tennis,” “Basketball,” “Skiing,” “Football,” “Badminton,” and “Boxing”). In contrast to conventional physical activities, interactive video games inherently engage children more actively, fostering their participation and enjoyment (Sun and Gao, 2016). These games provide a plethora of sensory stimuli, offering visually and auditorily enriching experiences (Stewart et al., 2020), while also enabling children to assume diverse roles and navigate varied scenarios within the game environment, thereby fostering curiosity and an inclination toward exploration (Zeng et al., 2020). At a mechanistic level, the anxiety-reducing effects of interactive video games bear similarities to physical activity. During gameplay, the release of neurotransmitters, including endorphins, in the child’s brain plays a pivotal role in mood regulation (Li et al., 2014). These neurotransmitters have been associated with enhanced emotional states, reduced anxiety symptoms, and the induction of feelings of happiness and relaxation (Santos Santos et al., 2021; Abd-Alrazaq et al., 2022). Furthermore, engaging in challenging and successful experiences during gameplay promotes a balance of neurotransmitters such as dopamine, norepinephrine, and serotonin, thereby enhancing children’s positive emotional experiences (Marques et al., 2023). It is noteworthy that the majority of game schemes employed in this study involve cooperative gameplay with two or more participants. This format of play, which emphasizes in-person interaction, provides children with a real social environment where they can engage with others in a secure and structured setting. When examining the impact of video game play on social interactions, it is important to distinguish between playing alone or online with peers and participating in face-to-face social play. Both types of play can promote social connections, but they may have different effects on social skills and emotional well-being. Research shows that in-person play often leads to richer social interactions because it allows for non-verbal communication cues, such as body language and eye contact, which are essential for developing interpersonal skills (Barman and Jena, 2023). In contrast, while virtual play offers opportunities to connect, it may lack these important cues, resulting in a different quality of interaction. For instance, studies have found that individuals who primarily communicate online report higher levels of social anxiety than those who engage in face-to-face interactions, indicating that a lack of in-person contact may hinder the development of vital social skills (Doorley et al., 2020). Moreover, playing video games in isolation can increase feelings of loneliness and disconnection (Sun et al., 2023). Conversely, multiplayer gaming can create a sense of community and shared purpose, even in a virtual setting. This suggests that while both virtual and in-person play have their advantages, the context of social interactions greatly affects their outcomes. Through gameplay, children are encouraged to collaborate towards shared objectives or interact based on common interests. This collaborative and interactive process not only enhances mutual communication among children but also cultivates their teamwork and problem-solving skills (Marlow et al., 2016; Yılmaz and Griffiths, 2023). Upon successfully achieving play objectives, children receive affirmative feedback from their peers, including praise and encouragement, which enhances their psychological assurance (Borowski et al., 2021) and mitigates feelings of self-doubt and anxiety commonly experienced in real social contexts. Future research should investigate whether interactive video game play that occurs virtually with others yields similar effects in reducing social anxiety. At the same time, interactive video games include more choices, which means endless possibilities.

LGT emerges as a significant intervention in reducing social anxiety levels among LBC. This discovery not only enhances our theoretical and practical understanding of psychological interventions for this demographic but also aligns positively with pertinent research involving autistic children. While previous studies primarily focused on the efficacy of LGT in enhancing children’s social skills (Legoff and Sherman, 2006; Lindsay et al., 2017), this study delves deeper into its favorable impact on alleviating social anxiety among LBC. LGT presents a novel and effective intervention for LBC. Within the game, each child assumes a specific role, possesses clear responsibilities, and follows instructions to collectively accomplish tasks. This mode of play effectively simulates a small social circle, facilitating the gradual adaptation and acclimatization of children to interaction with others within the game. Participation in LGT empowers LBC to not only enjoy cooperative endeavors but also gradually cultivate self-assurance and efficacy throughout task completion (Evans and Bond, 2021). Through these activities, they acquire essential communication, sharing, and problem-solving skills, thereby strengthening their interpersonal interactions in real-world scenarios. Furthermore, LGT underscores the stimulation of children’s creativity and imagination (Aksoy and Belgin Aksoy, 2023). Within the game, children construct an array of models tailored to their preferences and ideas, fostering innovative thinking and problem-solving abilities. Concurrently, collaborative efforts with peers foster a sense of teamwork and the joy of achievement, further enhancing their inclination towards social engagement and self-assurance.

The combined intervention demonstrated promising potential in reducing social anxiety among LBC by integrating the benefits of both IVG and LGT over a longer intervention period. These findings underscore the value of diverse, holistic approaches to mental health interventions that merge mind–body activities to support social engagement. While previous studies have often examined single interventions, such as either digital or physical play, these approaches may not fully address the multiple aspects of social anxiety. In this study, both IVG and LGT were utilized as structured social experiences that encouraged in-person interactions in varied settings. IVG provided opportunities for social skill practice through collaborative gameplay in a simulated environment, fostering initial comfort in peer interactions, while LGT emphasized hands-on interaction and the development of practical social skills. Rather than aiming to determine which method is superior, the combined approach in this study illustrates how different forms of social interaction can complement one another. The extended duration of the combined intervention may have also contributed to its effectiveness, as the sequential nature of IVG followed by LGT allowed children additional time to practice and reinforce social competencies. This approach may help bridge the gap between virtual and physical social settings, offering LBC varied opportunities to practice and strengthen social skills in ways that single interventions may not fully capture.

### Limitations and future directions

Firstly, this study used self-report scales to measure social anxiety in LBC. While self-reports can gather a lot of information, they may be influenced by personal biases, which could affect accuracy. Future research could include physiological measures to provide more objective data on children’s reactions to social situations. Secondly, the study used a single-blind design, where the researcher was unaware of certain details but the participants were not. This could lead to participants being influenced by expectations when filling out the self-report scales. Future studies should consider a double-blind design to reduce these biases. Thirdly, another limitation is the different task complexities in the LGT intervention. While all groups had the same overall building goals, the specific tasks were tailored to each group’s developmental level. This could affect how comparable the results are between groups. Future research should use standardized tasks or account for developmental differences more carefully. Fourthly, it is essential to acknowledge the inherent limitations in comparing the Lego Therapy and IVG interventions. These interventions differ significantly in their nature, promoting either physical or digital play, and in the degree to which they focus on building versus physical movement. Additionally, the assignment of social roles within Lego Therapy creates unique social dynamics that are not present in the IVG intervention. These differences make it challenging to determine which intervention is more effective at decreasing social anxiety and under what circumstances. Furthermore, the Combination Intervention presents additional challenges in comparison. With over double the intervention time of the other two approaches, it is unclear whether its efficacy stems from the synergistic effect of combining both types of play or simply from the increased duration of intervention. These factors must be considered when interpreting the results, as they could significantly impact the outcomes. Moreover, there was also a difference in participation time between the IVG and LGT. In the IVG, children played in pairs, so not everyone was actively engaged for the full 45 min. In contrast, all participants in the LGT were involved throughout the session. This difference in engagement time could have impacted the results, so future research should create more balanced participation structures or examine how different levels of engagement affect outcomes. A key limitation of this study was the lack of counterbalancing between the order of the IVG and LGT interventions. Participants either experienced IVG or LGT first, which might have affected their experiences in the second intervention. Without counterbalancing, observed effects could be influenced by the order of interventions. Future studies should use a counterbalanced design to control for this potential bias. Finally, the study was based on a small group of LBC from one city in China. This raises concerns about how the findings might vary in different regions or cultures. Future research should aim to include a larger and more diverse sample of LBC to enhance the generalizability of the results.

## Conclusion

This study explored the impact of IVG and LGT on reducing social anxiety in LBC, with a focus on how different forms of social interaction might contribute to alleviating anxiety symptoms. Findings suggest that both IVG and LGT offer significant benefits in reducing social anxiety, with the combination intervention showing additional promise by integrating the strengths of both approaches. The results underscore the value of diverse, holistic approaches to mental health interventions, highlighting the rehabilitative potential of combining virtual and hands-on social experiences as a therapeutic strategy for this population’s mental health needs. Importantly, each of the individual interventions demonstrated efficacy, suggesting that either IVG or LGT can be a viable option for reducing social anxiety in LBC. Encouraging the adoption of both standalone and combined intervention strategies could enhance clinical practice for addressing social anxiety in LBC. Furthermore, adapting these interventions to consider the unique circumstances and cultural backgrounds of specific regions could improve their relevance and effectiveness, ensuring that the approach aligns with the distinct needs of LBC across diverse geographical settings.

## Data Availability

The raw data supporting the conclusions of this article will be made available by the authors, without undue reservation.
